# Long COVID Syndrome and Associated New Daily Persistent Headache (NDPH) Presenting With Crossed Clonus Response

**DOI:** 10.1002/ccr3.72386

**Published:** 2026-03-20

**Authors:** Emma Wetmore, Stephanie Grach, Christopher Boes, Giuseppe Lanzino, Narayan Kissoon

**Affiliations:** ^1^ Department of Neurology Mayo Clinic Rochester Minnesota USA; ^2^ Department of General Internal Medicine Mayo Clinic Rochester Minnesota USA; ^3^ Department of Neurosurgery Mayo Clinic Rochester Minnesota USA

**Keywords:** crossed clonus, long covid, meningioma, neurology, new daily persistent headache

## Abstract

Evaluating patients following a COVID‐19 infection with multiple chronic, progressive symptoms can be challenging, but easy to navigate by using the temporal profile obtained from the clinical history, findings from the neurological exam, and screening for red flag symptoms.

## 1 Introduction

Long COVID is a clinical diagnosis. According to the NASEM 2024 criteria, symptoms must be present for at least 3 months following COVID‐19 diagnosis with symptom onset either immediately after diagnosis or delayed up to months; additionally, symptoms can be mild or severe, can be constant, intermittent, or progressive, and can last for months to years [1]. The most frequent symptoms are post‐exertional malaise, fatigue, brain fog, dizziness, and gastrointestinal symptoms [2]. Given the wide range of conditions that can occur after COVID, Long COVID specialists are increasingly using the term “syndromic Long COVID” to specifically refer to symptoms suggestive of or meeting criteria for infection‐associated chronic conditions like myalgic encephalomyelitis/chronic fatigue syndrome and postural orthostatic tachycardia syndrome, including fatigue, brain fog, sleep disruption, and orthostatic intolerance not otherwise explained by another condition.

## 2 Case History/Examination

A 72‐year‐old male presented to their local neurologist with a 16‐month history of a new daily persistent headache (NDPH) following a COVID‐19 infection. The headache was accompanied by cognitive symptoms, non‐vertiginous dizziness, and lower extremity paresthesias. Prior to the infection, he received 2 doses of the Pfizer COVID‐19 vaccine, and at the time of diagnosis he received no acute therapies. Past medical history was significant for prostate cancer (diagnosed in 2011) status post radiation, colon cancer (diagnosed in 2016) status post laparoscopic partial colectomy, chronic kidney disease with a baseline creatinine of 1.5, depression, mild obstructive sleep apnea (not on positive airway pressure therapy), coronary artery disease (on atorvastatin 40 mg), migraine (on topiramate 50 mg BID), and prior concussions (at age 3 secondary to a fall, and at age 13 secondary to sport).

The patient's headache had a distinct day of onset following COVID‐19 infection, with no moments of headache freedom thereafter. He described his headaches as severe, throbbing, worse with exertion, and associated with phonophobia only (not photophobia or nausea). Symptoms were accompanied by a gradual worsening in forgetfulness and difficulties multitasking, restless leg symptoms, and numbness and tingling in the feet. The paresthesias involved the bilateral feet circumferentially, with the left affected more than the right. He also endorsed a recent psychiatric hospitalization 2 months prior for suicidal intent. He denied falls but endorsed increased dizziness when bending down or turning. He denied hearing loss.

Physical exam findings at this time were notable for symmetric 3+ reflexes (NINDS myotatic reflex scale [MRS]), non‐sustained clonus in the left ankle, and some difficulty with tandem walking. His Montreal Cognitive Assessment (MoCA) was 26/30. At the time, memory difficulties were suspected to be related to pseudodementia from depression. MRI of the lumbar spine showed multilevel spondylotic changes including moderate left L5‐S1 neural foraminal stenosis, mild right L5‐S1 neural foraminal stenosis, mild bilateral neural foraminal stenosis L4‐5, and mild central canal stenosis at L4‐5 and L5‐S1. With these radiographic findings, the foot paresthesias were attributed to lumbosacral radiculopathies by his local providers, and he was treated with physical therapy. For the headaches, he was started on propranolol 60 mg for migraine prevention and sumatriptan as an abortive medication.

He was referred to a post‐COVID clinic about 22 months following his positive COVID‐19 test due to his persistent symptoms. At this appointment, he noted a persistent, progressive worsening of fatigue, brain fog (manifesting as difficulty concentrating/remembering and difficulty driving), and headache. Over time, he also developed insomnia (treated with nightly trazodone 200 mg), tinnitus, dizziness with poor balance, depression, and new incontinence. He described his dizziness as occurring at all times and worsened with climbing stairs and bending over. He reported exercise intolerance secondary to worsened generalized weakness and energy, and that he was now unable to ride his bicycle or drive due to a new tendency to veer to the left. He also reported that his left foot “flops.”

He was then referred to a headache specialist, at which time his physical exam was notable for preserved strength on motor exam, but clear asymmetry in reflexes with a Hoffmann sign on the left, sustained clonus in the left ankle that was also provoked when eliciting a right plantar reflex (crossed clonus response), moderately increased reflexes in the left biceps, brachioradialis, and quadriceps, and significantly increased reflexes in the left gastroc‐soleus with associated clonus.

## 3 Differential Diagnosis, Investigations and Treatment

The patient described above presented with symptoms (e.g., headache, fatigue, dizziness) that are often described in Long COVID syndrome and NDPH. However, the progressive worsening of symptoms and focal neurological signs would warrant considerations for an alternative diagnosis. The cognitive symptoms and headache could suggest an intracranial central nervous system lesion. The reflex changes would localize to the right cerebral hemisphere. The urinary incontinence would suggest a lesion affecting the periventricular white matter and the neuropsychiatric symptoms could potentially localize to the right orbitofrontal region or a global cortical process. With the relatively preserved strength on manual motor testing, the descriptive symptoms of left‐sided weakness with veering to the left would suggest either apraxia or hemispatial neglect. Cerebellar pathology was less likely in the absence of cerebellar signs such as ataxia, dysmetria, or nystagmus. The crossed clonus finding on exam would suggest a large lesion affecting both cerebral hemispheres. The lower extremity paresthesias were in a non‐dermatomal pattern, but in the context of the observed reflex changes, could localize to the primary sensory cortex adjacent to the interhemispheric fissure. The mild to moderate spondylotic changes noted on the MRI of the lumbar spine did not explain these symptoms.

There are several alarm symptoms and signs in this presentation, and the SNOOP mnemonic can be used to identify these features (Systemic symptoms/signs, Neurologic symptoms/signs, Onset sudden, Older age of onset, Pattern change) [3]. In this presentation, the prior history of malignancy, neurologic signs on exam with left sided hyperreflexia and crossed clonus response, NDPH presentation, and age of onset at 70 are all alarm features to suggest a secondary cause to the headache.

## 4 Conclusion and Results (Outcome and Follow‐Up)

**FIGURE 1:**
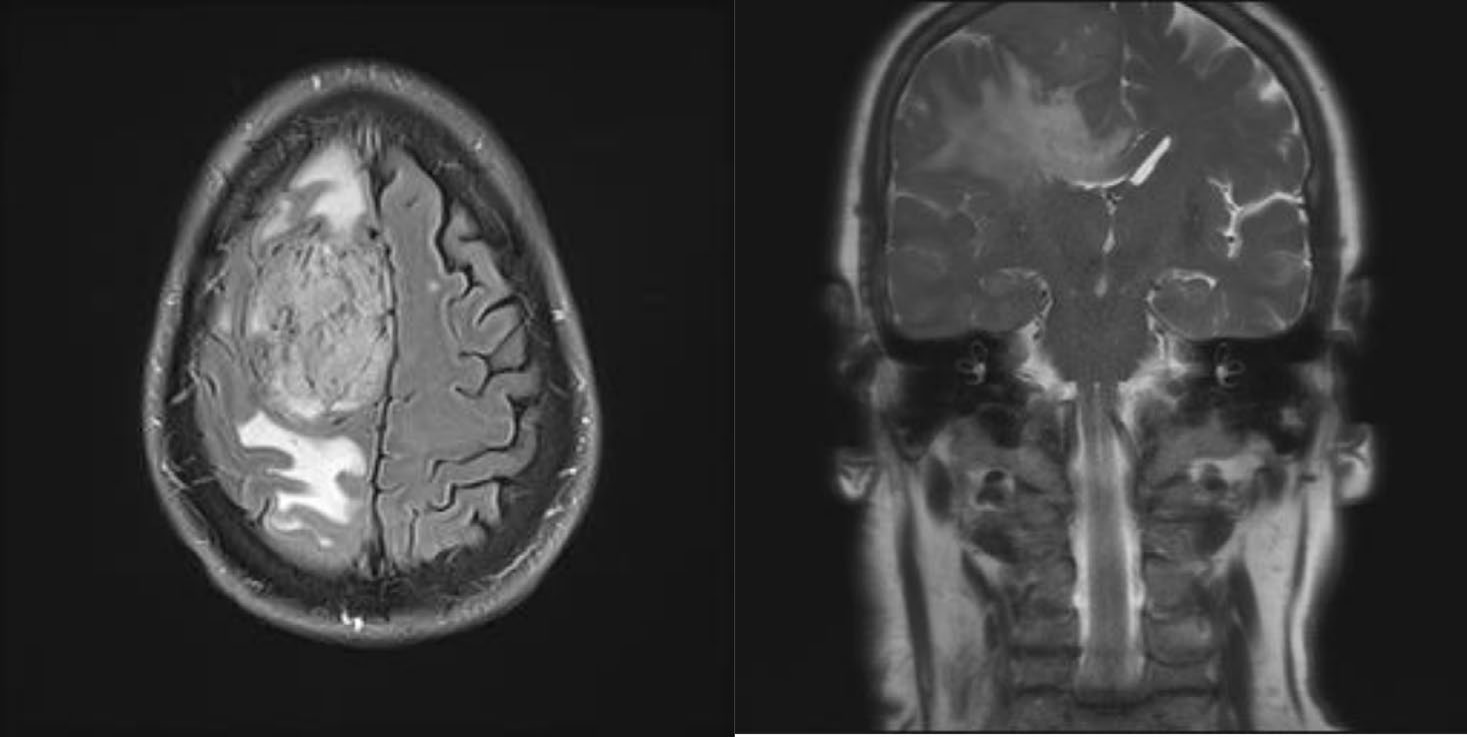
Extra‐axial mass arising from the right parasagittal calvarial vertex measuring 6.1 cm by 3.5 cm by 4.8 cm with leftward subfalcine herniation of 11 mm and partial effacement of the right lateral ventricle. There is surrounding vasogenic edema with extension into the corpus callosum and no significant transtenorial (uncal) herniation.

The patient described above was referred for an MRI brain, which was significant for a large extra‐axial mass measuring 6.1 cm by 3.5 cm by 4.8 cm with leftward subfalcine herniation of 11 mm and partial effacement of the right lateral ventricle (Figure 1). He was ultimately taken for surgery, which revealed a stage 2 meningioma for which he had a near‐total resection and adjuvant radiation therapy. Following surgical resection, headaches resolved for a period of time; however, this was followed by recurrent headaches without tumor recurrence on subsequent imaging. Fatigue and insomnia improved with the patient overall feeling about 85% better 6 months following resection. The response to treatment of the meningioma further supported that the headaches were secondary to mass effect from the meningioma rather than syndromic Long COVID or NDPH.

## 5 Discussion

As it pertains to headache, the clinical phenotype in this case resembled new daily persistent headache (NDPH), which typically follows a systemic viral illness. The International Classification of Headache Disorders (ICHD) version 3 does indicate that the diagnosis for NDPH should be made when symptoms are not better accounted for by another ICHD‐3 diagnosis [4]. In this case, presence of focal neurologic deficits points away from NDPH and towards a secondary process. Interestingly, post‐infectious syndromes and NDPH have been described in prior pandemics (e.g., 1890 Russian/Asiatic Flu viral pandemic) [5]. Until there is another update to the ICHD, the symptom of a new daily headache lasting at least 3 months as a part of Long COVID should be classified as NDPH per ICHD‐3 criteria rather than “chronic headache attributable to systemic viral infection,” which would require an active viral infection or resolution within the prior 3 months.

The signs and symptoms that have localizing features that could be attributed to the meningioma include reflex changes, incontinence, and descriptive symptoms of veering to the left, which are suggestive of apraxia or hemispatial neglect. The non‐localizing symptoms, which include headaches, cognitive symptoms, and dizziness, could be attributable to both Long Covid and the meningioma affecting the right cerebral hemisphere. The significance of the restless leg symptoms and bilateral foot paresthesias is unclear and potentially could not be attributable to either disorder.

It is possible that the bilateral foot paresthesias could be related to the meningioma.

Affecting the primary sensory cortex adjacent to the interhemispheric fissure, but this would be speculative.

## 6 Crossed Clonus Response

The crossed clonus response observed in this case was recently reported in a prior patient with cervical myelopathy, and the presence would suggest a large lesion affecting bilateral pyramidal tracts [6]. The crossed clonus sign is likely a result of a lesion affecting both cerebral hemispheres and may be akin to the finding of Kernohan‐Woltman notch on pathologic specimens with transtentorial (uncal) herniation [7, 8]. However, transtentorial herniation was not observed on the imaging in this case, so the crossed clonus response was likely related to dysfunction of the interneuronal pathways crossing the corpus callosum (Figure 1) [9].

## 7 Surveillance

**FIGURE 2:**
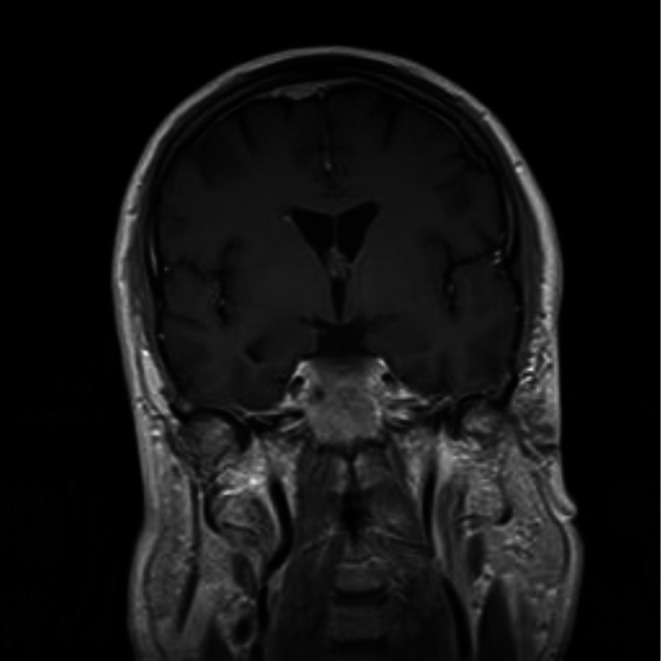
Small right parasagittal contrast‐enhancing dural based lesion observed 4 years prior to symptom onset consistent with meningioma.

In retrospect, prior imaging from 4 years prior to symptom onset demonstrated a small enhancing dural based right parasagittal lesion in the same location as the later demonstrated meningioma (Figure 2). While there are no formal guidelines to direct the interval and duration of monitoring for incidental meningiomas, this case also highlights the importance of surveillance imaging for meningiomas, especially if there is a clinical change that dictates repeat imaging [10].

In summary, this case report shows the importance of not anchoring on one diagnosis and how the neurological exam, temporal profile, and screening for red flag symptoms should guide management of headache.

## Author Contributions

**Emma Wetmore:** data curation, investigation, writing – original draft. **Stephanie Grach:** investigation, writing – review and editing. **Christopher Boes:** investigation, writing – review and editing. **Giuseppe Lanzino:** investigation, writing – review and editing. **Narayan Kissoon:** conceptualization, investigation, writing – review and editing.

## Funding

The authors have nothing to report.

## Consent

Verbal and written consent from the patient was obtained for this case report to be written. Written informed consent from the patient was obtained according to journal guidelines.

## Conflicts of Interest

The authors declare no conflicts of interest.

## Data Availability

Data available on request from the authors.
